# Targeting hexokinase 2 for oral cancer therapy: structure-based design and validation of lead compounds

**DOI:** 10.3389/fphar.2024.1346270

**Published:** 2024-03-11

**Authors:** Purbali Chakraborty, Syeda Lubna, Shouvik Bhuin, Deepika K., Manab Chakravarty, Trinath Jamma, Perumal Yogeeswari

**Affiliations:** ^1^ Department of Pharmacy, Birla Institute of Technology and Science, Hyderabad, India; ^2^ Cancer Research Group, Centre for Human Diseases Research, Birla Institute of Technology and Science, Hyderabad, India; ^3^ Department of Biological Sciences, Birla Institute of Technology and Science, Hyderabad, India; ^4^ Department of Chemistry, Birla Institute of Technology and Science, Hyderabad, India

**Keywords:** oral cancer, hexokinase-2 inhibitor, pharmacophore, molecular dynamics, structure-based, mitochondria membrane potential, spheroids

## Abstract

The pursuit of small molecule inhibitors targeting hexokinase 2 (HK2) has significantly captivated the field of cancer drug discovery. Nevertheless, the creation of selective inhibitors aimed at specific isoforms of hexokinase (HK) remains a formidable challenge. Here, we present a multiple-pharmacophore modeling approach for designing ligands against HK2 with a marked anti-proliferative effect on FaDu and Cal27 oral cancer cell lines. Molecular dynamics (MD) simulations showed that the prototype ligand exhibited a higher affinity towards HK2. Complementing this, we put forth a sustainable synthetic pathway: an environmentally conscious, single-step process facilitated through a direct amidation of the ester with an amine under transition-metal-free conditions with an excellent yield in ambient temperature, followed by a column chromatography avoided separation technique of the identified lead bioactive compound (**H2**) that exhibited cell cycle arrest and apoptosis. We observed that the inhibition of HK2 led to the loss of mitochondrial membrane potential and increased mitophagy as a potential mechanism of anticancer action. The lead **H2** also reduced the growth of spheroids. Collectively, these results indicated the proof-of-concept for the prototypical lead towards HK2 inhibition with anti-cancer potential.

## 1 Introduction

Oral cancer holds the unfortunate distinction of being the sixth most prevalent form of cancer worldwide. India reports highest numbers of oral cancer cases in the world with a rapid increase in annual incidence and mortality (Sharma et al., 2018; Mathur et al., 2020). The chemotherapeutic drugs that are administered in combination with radiation or as a standalone cancer therapy of the oral cavity and oropharynx include cisplatin, carboplatin, 5-fluorouracil, paclitaxel, docetaxel, methotrexate, capecitabine and hydroxyurea (Olivier et al., 2021; Sochacka-Ćwikła et al., 2022). Predominant targeted monoclonal antibody therapy encompasses cetuximab (anti-EGFR antibody) to treat cancer (Liu et al., 2019). Nevertheless, no small molecule targeted drug has been in the clinic for standalone oral cancer therapy so far, as drug resistance and toxicity pose considerable concerns while providing scope for further exploration (Wen and Grandis, 2015; Kordbacheh and Farah, 2021). Previous decades have seen the usage of drug combinations like carboplatin-paclitaxel and docetaxel-cisplatin-fluorouracil as chemotherapeutic agents (Agarwala et al., 2007). Several multi-kinase inhibitors in combination with conventional chemotherapies are the current therapeutic strategies for oral and oropharyngeal cancers (Brands et al., 2019; Dusi et al., 2019). Epidermal growth factor receptor tyrosine kinase inhibitors (EGFR-TKIs) like gefitinib, erlotinib and lapatinib (EGFR-TKIs) are administered with chemotherapeutic agents to enhance their sensitivity in head and neck squamous cell carcinoma (HNSCC) (Tang et al., 2019; Yang et al., 2021). Further, multi-kinase inhibitors (sorafenib, pazopanib and dovitinib) have also shown enhanced therapeutic efficacy when administered with cisplatin in HNSCC cell lines targeting angiogenesis and metastasis (Lo Nigro et al., 2017; Chen and Hong, 2022).

For decades, inhibition of glycolysis has been a therapeutic approach for anti-cancer treatment (Bai et al., 2021; Liu et al., 2021; Chakraborty et al., 2022). Hexokinase, the first-rate limiting enzyme of the glycolytic pathway catalyzes the conversion of glucose to glucose-6-phosphate thus, contributing to the “Warburg effect” (increase in the glucose uptake rate and preferential production of lactate even under normoxia) (Fontana et al., 2022; Sruthi and Raghu, 2022). There are five major hexokinase isoenzymes (HK1, HK2, HK3, HK4, and HKDC1 (hexokinase domain containing protein 1), and a major portion of intracellular HK2 lies in proximity to mitochondria stabilizing the mitochondrial membrane potential (MMP) in association with voltage-dependent anion channel (VDAC) to prevent the release of cytochrome *c* and inhibiting apoptosis (Pastorino et al., 2002; Fan et al., 2019; Ciscato et al., 2020). Notably, HK2 is overexpressed in many aggressive tumors and localized to mitochondrial outer membrane attaining higher conformational stability compared to cytosolic HK2 (Pedersen et al., 2002; Anderson et al., 2017) suggesting, HK2 as a novel target for drug discovery against a multitude of cancers. However, it comes with its own challenges such as highly polar nature of HK2 active site and its lower affinity to interact with VDAC compared to that of HK1, altogether complicating the drug discovery process (Heslop et al., 2021; Zheng et al., 2021; Shan et al., 2022).

Mitophagy, the selective degradation of damaged mitochondria, plays a pivotal role in maintaining cellular homeostasis. It is intricately linked to the loss of mitochondrial membrane potential (MMP), a key indicator of mitochondrial dysfunction. This decline in MMP serves as a signal for the initiation of mitophagy, leading to the removal of impaired mitochondria and prevention of their accumulation within cells (Pickles et al., 2018). The hit compounds outlined in this paper have a variety of scaffolds (pharmacophores) which were obtained from a multiple pharmacophore-based high throughput virtual screening campaign using Schrodinger software modules for drug discovery employing various HK2 proteins (Liu et al., 2020; Zhang et al., 2021; Deng et al., 2022). Upon examining the efficacy of the compounds against HK2 in different cell-based assays, we found a benzothiazole-carboxamide compound (**H2**) and a benzothiazole-methoxyphenyl urea compound (**H10**) inhibiting HK2 enzyme activity. Interestingly, our research group has previously recognized these compounds as potential EGFR-TK inhibitor candidates for the treatment of oral cancer (Chakraborty et al., 2022). The convergence of hit compounds identified in both our earlier research and the present study signifies an expanded opportunity for discovering prototype lead compounds targeting cellular kinases. Upon carrying out additional proof of concept experiments, it became evident that compound **H2** performed better over the benzothiazole-urea derivative (**H10**) in the *in vitro* assays using FaDu and Cal27 oral cancer cell lines. After validating the anti-cancer potential of the HK2 inhibitors, mechanistic studies were carried out employing **H2** in the relatively sensitive Cal27 cell line. The studies have conclusively showed that **H2** is a potent inhibitor of HK2 enzyme activity and an inducer of mitophagy which holds promise for developing effective therapies for oral cancer.

## 2 Materials and methods

### 2.1 Synthetic approaches of the lead HK2 inhibitor

The synthetic procedure, along with a plausible mechanism and comprehensive characterizations, is provided in the Supporting Information.

### 2.2 Computational approaches

Molecular docking studies were performed using Glide module (Schrödinger Maestro 2022), Ligplot module was used to prepare ligands, the pharmacophore features were developed using Phase module in Maestro. MD simulations were performed using Desmond module and the binding energy calculations were performed using MM-GBSA module.

#### 2.2.1 Protein structure preparation, hypothesis generation for EP and validation of E-pharmacophores and screening of database

The crystal structures of HK2 protein bound with inhibitors were retrieved from RCSB PDB (PDB ID: 2NZT, 5HEX, 5HG1 and 5HFU). The protocol for protein preparation, the EP hypothesis generation protocol, the protocol for validation of EP and molecular docking have been described previously (Chakraborty et al., 2022).

#### 2.2.2 MD simulations and calculation of MM-GBSA

To study the binding affinity of our proposed lead compound, MD simulations were performed for five different systems, 1CZA (HK1) and 5HEX (HK2) complex structure with their co-crystal ligands, and 1CZA and 5HEX with our modeled ligand (**H2**), and 1CZA with the co-crystal ligand of 5HEX. The top ranked docked poses were taken forward for performing MD simulations. The MD simulations were carried out under explicit water solvent. The enzyme-ligand complex was immersed in a cubic box of TIP3P water molecules with a dimension of 10 Å using System Builder tool and OPLS3e was chosen as the force field to build the systems. Simulations for all the systems were carried out under NPT canonical ensemble for 100 ns each with constant temperature of 310 K and constant pressure at 1.01325 bar. The trajectories were saved at every 10 ps. Data were analyzed using simulations interaction diagram (SID) and binding energy was calculated for the enzyme ligand complex using the Prime MM-GBSA module of Schrödinger.

### 2.3 Cell culture

FaDu cell line was cultured in RPMI-1640 medium containing L-glutamine (300 mg/L) and sodium bicarbonate (2 g/L), while Cal27, HEK293 and HFF-1 cell lines were cultured in DMEM medium containing glucose (4.5 g/L), L-glutamine (584 mg/L) and sodium bicarbonate (3.7 g/L). For gene expression and qPCR studies, RNA isolation was performed using Trizol (Thermo). The reagents and the primer sequences employed for the qPCR assays have been described previously (Chakraborty et al., 2022).

### 2.4 HK2 enzyme inhibition assay

The HK2 enzyme inhibition assay was performed using an HK2 inhibitor assay kit (Abcam, ab211114) according to the manufacturer’s instructions. Initially, 50 µM compound concentration was used to perform the enzyme activity inhibition studies, after which IC50 of the top leads were determined.

### 2.5 Flow cytometry based glucose uptake assay and mitochondria membrane potential analysis

FaDu and Cal27 cells were treated with test compounds (0.2 million cells per treatment) and were incubated for 1 h with 100 μM of 2-NBDG (Invitrogen, N13195), in glucose free medium, washed with ice-cold PBS, and re-suspended in 500 μL of PBS and maintained on ice to perform flow cytometric analyses within 30 min. The mean fluorescent intensity (MFI) readout reflects the amount of fluorescent glucose analog taken up by the cells, providing insight into their glucose uptake capacity. The percent (%) cell population positive for glucose uptake (shown with a right shift of the histogram peaks) were also plotted for both the cell lines. For MMP analysis, cells were incubated with 200 nM of TMRE (Invitrogen, T669) for 30 min. For each measurement, data was collected for 10,000 events using a BD FACS Aria III (Becton Dickinson, San Jose, CA) instrument.

#### 2.5.1 Cell cycle analysis and apoptosis assay

FaDu and Cal27 cells were treated with the test compounds (0.5 million cells per treatment) for 72 h, and harvested for cell cycle analysis (Cat# BD340242) and apoptosis analysis (Cat# BD556547) by flow cytometry according to manufacturer’s instructions.

### 2.6 Western blotting

Cal27 cells were treated with the test compound (1 million cells per treatment for 4 h) and were processed for mitochondria isolation using a homogenization buffer containing 210 mM mannitol, 70 mM sucrose, 5 mM tris-HCl, (pH 7.5) and 1 mM EDTA (pH 7.5). The cell pellet was sheared with a total of 90 strokes in the homogenization buffer in a span of 1 h on ice. The lysate was centrifuged at 800 rpm for 10 min and the mitochondria enriched supernatant was isolated. The pellet was again lysed using RIPA lysis buffer to obtain the cytosolic fraction. The mitochondrial fractions and the cytosolic fractions were loaded on a 10% SDS-PAGE gel. The antibodies used were anti-HK2 antibody (CST, Cat#2867), anti β-actin antibody (CST, Cat#4970) and mitochondrial marker anti-VDAC antibody (CST, Cat# 4661). The whole cell lysates were obtained using RIPA lysis buffer alone. The quantifications were performed using ImageJ software.

### 2.7 Immunofluorescence

Cal27 cells were treated with respective compounds for 4h, and washed with PBS and fixed with 100% methanol. The non-specific antibody binding was blocked by incubating with 5% goat serum in 0.3% Triton X-100 for 1 h. The diluted primary antibody (LC3B mAb, CST Cat# 3868) and MitoTracker Red (CST, Cat# 9082) were applied and samples were incubated overnight at 4°C followed by addition of fluorescently labelled secondary antibody for 2 h at room temperature and visualized using Leica DMi8 laser scanning confocal microscope using the Leica LAS X software. To analyze the extent of mitophagy in the captured images, the colocalization area of green (LC3B) and red (Mito-Red) portions were quantified using the LAS X software. The LC3B MFI was also quantified and represented in gray values.

### 2.8 3D spheroid culture, flow cytometry analysis of spheroids and analysis of spheroid images

Cal27 cells were seeded at a density of 10,000 cells per well in a 96-well plate coated with 1.5% agarose in incomplete medium. For the prophylactic treatment, medium containing the compounds was added to each well after 6 h of seeding. For treatment, compounds were added after 48 h of seeding. After incubation with the compounds, spheroids were imaged using phase-contrast microscopy to assess the morphology and size. Subsequently, the spheroids were pooled and dissociated into single cell suspension with trypsin for 10 min, washed with PBS and stained with propidium iodide (Cat# BD556547) to analyze the number of apoptotic cells using flow cytometry. For the analysis of spheroid images, each image was processed using ImageJ software where the threshold of each 8-bit image was adjusted to differentiate the darker spheroid area (the inner core) from the lighter spheroid area (the peripheral layers). Subsequently, the darker and lighter areas were measured in pixel square units using the ‘analyze particles’ option in ImageJ.

### 2.9 Statistical analyses

Statistical analyses were performed using GraphPad Prism 8.0 software. Data are presented as mean ± SD of individual sets of experiments. The number of replicates (n) for each experiment have been mentioned in the figure captions. Statistical significance was assessed using Student’s t-test for comparisons between two groups and one-way ANOVA followed by Dunnett’s test for multiple group comparisons. A *p*-value less than 0.05 was considered statistically significant.

## 3 Results

### 3.1 Structure-based drug design for HK2 inhibitors

**FIGURE 1:**
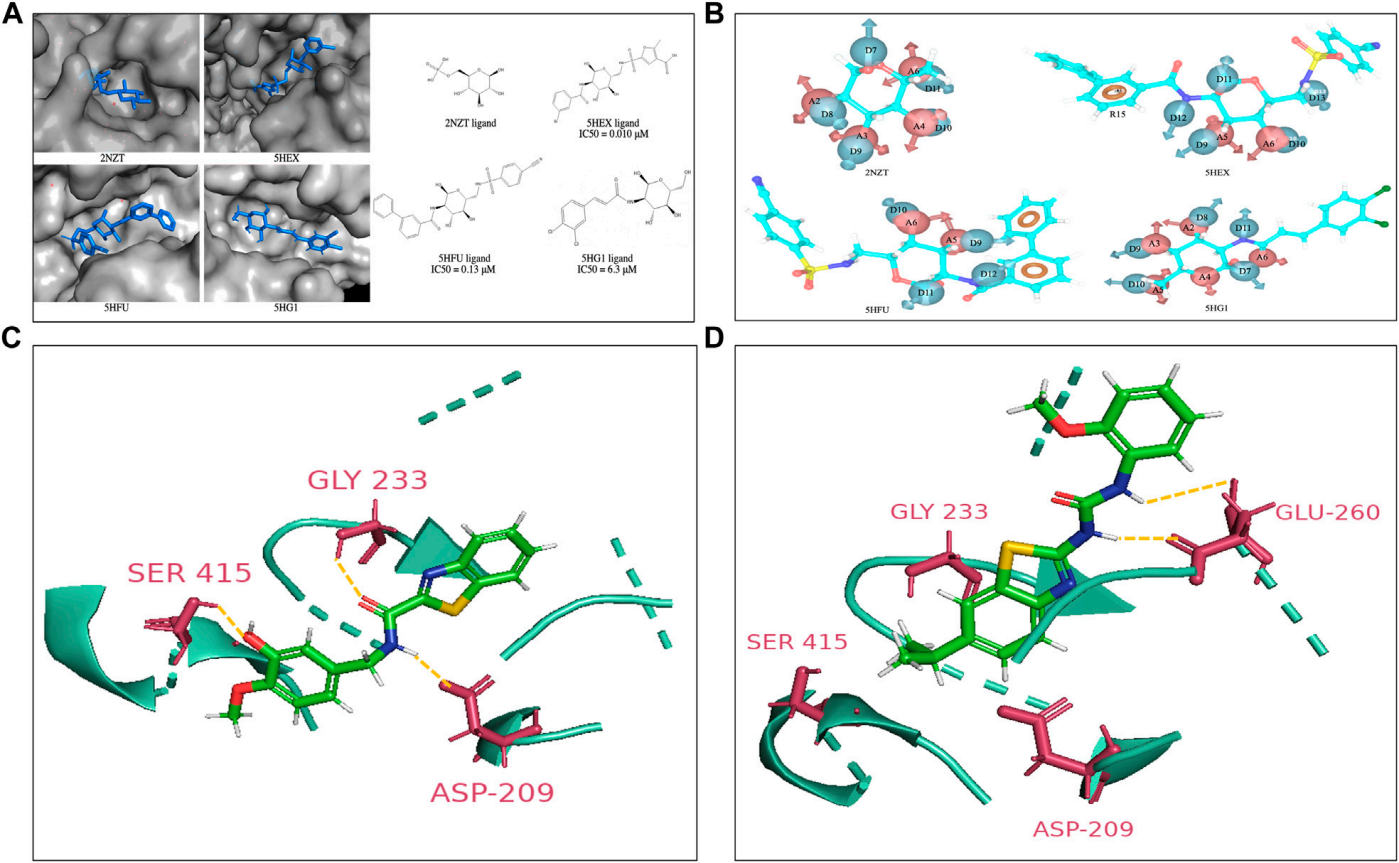
**(A)** Co-crystal structures of the HK2 proteins with indicated ligands in the substrate binding pocket. The 2D structures of respective crystal ligands with their reported IC_50_ values are also presented **(B)**. The energy-based pharmacophore hypotheses generated from structure-based modelling showing the crystal ligands of HK2 proteins **(C)**. Lead compound at active site of HK2 enzyme. Compound **H2** at the glucose binding domain of HK2 protein shows H-bond interactions with residue Asp209, Gly233 and Ser415 **(D)**. Compound **H10** at the glucose binding site of HK2 protein shows H-bond interactions with residue Glu260. The interacting residues have been highlighted in yellow.

Structure-based and ligand-based drug design strategies have been employed for re-purposing of the drugs (Castleman et al., 2022; Chakraborty et al., 2022). The only available crystal structures of HK2 protein in complex with co-crystal ligands in RCSB Protein Data Bank {PDB IDs: 2NZT (2.45 Å), 5HEX (2.73 Å), 5HFU (2.92 Å) and 5HG1 (2.76 Å)} suggest that inhibitors have been designed towards the substrate (glucose) binding site (Figure 1A). The Kaplan Meier overall survival analyses (performed on datasets from the TCGA of head and neck cancer patients in UCSC Xena) characterize HK2 expression (both low/high) and associate with significantly poor patient overall survival, thus rendering HK2 as an attractive target (Supplementary Figure S1). Earlier reports show that glucosamine derivatives have exhibited higher potency in HK2 inhibition with higher selectivity towards HK2 over HK1 (Lin et al., 2016). However, the highly polar active site of HK2 and affinity towards other HK isoforms make the discovery process complex. Therefore, we put forward hypothesis derived from the EP (energy-based pharmacophores) generated from the co-crystal ligands of the protein crystal structures as shown in Figure 1B, to design inhibitors against HK2. The co-crystal ligands from 2NZT, 5HEX, 5HFU, and 5HG1 were used to generate a total of 55 hypothesis by combining different parameters of the main hypothesis as shown in Figure 1B. The selection of compounds for hypothesis generation was not based on a specific pIC50 cutoff. Instead, all four co-crystal ligands obtained from available HK2 crystal structures from the RCSB PDB were used in our study. All of the 55 hypotheses were validated using enrichment and BEDROC statistical calculations. Among them, 27 hypotheses were found valid as the model could filter out the active molecules from a set of active and decoy ligands (Table S1). The valid EPs were then used to screen the ligand library for similar compounds using the Phase module. The library consisted of US-FDA-approved drugs and compounds synthesized in our lab setting (BITS in-house database), incorporating 5998 ligands. Further, the Phase hits were re-docked to the active site of HK2 protein following a virtual screening campaign (HTVS, SP, and XP docking), which yielded a set of hits for further screening.

### 3.2 Evaluation of antiproliferative activity of inhibitors and HK2 enzyme inhibitory activity

The hits derived from the docking study were sourced from either commercial suppliers or pre-existing compounds within our laboratory or were synthesized anew. The FaDu and Cal27 oral cancer cells were treated with the compounds for 72 h at concentrations of 3, 10, 30, and 100 μM. A post-treatment MTT assay was done to evaluate their antiproliferative activity. Table S2 represents the anti-proliferative and enzyme inhibitory activity of the compounds obtained as hits from the structure-based drug design strategy. The standard HK2 inhibitor 2-deoxyglucose (2-DG, a d-glucose mimic) showed a GI_50_ of 77.56 μM in Cal27 cells with no efficacy against FaDu cells. On the other hand, 3-bromopyruvate (3-BP) exhibited a GI_50_ of 36.36 μM and 23.85 μM in Cal27 and FaDu cells, respectively. Consequently, 50 μM of all the hit compounds were evaluated for their HK2 enzyme inhibitory activity. 50 μM of compounds were incubated with HK2 protein and the rate of reaction was assessed in kinetic mode (rate of conversion of NAD to NADH). The choice of 50 μM concentration was based primarily on MTT assays and GI50 calculation. The average GI50 values of the hit compounds against less sensitive FaDu cell line was approximately 50 μM. Considering this information, we reasoned that utilizing a concentration equivalent to the average GI50 would allow us to assess the maximum inhibitory effect of the compounds on HK2 enzyme activity. We found that only compounds 11 and 14 showed HK2 inhibition of 82.8% and 87% at 50 μM concentration. The same compounds have been previously reported by our team to have EGFR phosphorylation inhibitory potential and were coded as **H2** (compound 14) and **H10** (compound 11) (Chakraborty et al., 2022). The IC_50_ values of **H2** and **H10** against HK2 were 2.94 ± 0.2 µM and 13.89 ± 0.5 µM, respectively, whereas the IC_50_ of 3-BP as reported against HK2 is < 5 µM (Liu and Liu, 2022). None of the molecules which showed better growth inhibition against FaDu and Cal27 had any HK2 inhibitory efficacy except **H2** and **H10**. The GI_50_ of **H2** and **H10** against FaDu were 63.06 μM and 65.92 μM, respectively, whereas the GI_50_ of **H2** and **H10** against Cal27 were 21.70 μM and 28.29 μM, respectively, suggesting the sensitivity of Cal27 towards the designed HK2 inhibitors. Figures 1C,D show the lead compounds **H2** and **H10** at the active site of HK2 enzyme interacting via H-bonds with the amino acid residues.

### 3.3 Molecular dynamics simulations and binding energy calculations

Following the enzyme inhibition assays, molecular dynamics (MD) simulations were conducted using the Desmond package. The objective of conducting MD simulations was to gain insights into the interaction patterns between the lead compound and HK1 and HK2 proteins over a 100 ns period. This analysis was crucial for assessing the lead compound’s selectivity for HK2 over HK1. The protein root mean square deviation (RMSD) for 5HEX exhibited similar changes in both MD simulation scenarios: one with ligand **H2** and the other with co-crystal ligand 604. This illustrates the stability of the HK2 protein in the presence of the lead compound, as evidenced by the co-crystal ligand RMSD displaying a slightly higher variation for optimal fitting into the binding pocket (Supplementary Figures S2B, D). In contrast, during MD simulations of HK1 (1CZA) with ligand **H2**, both the protein and ligand **H2** exhibited higher RMSD fluctuations (Supplementary Figures S2A, C). This implies the greater stability of ligand **H2** at the active site of HK2 compared to HK1. Examining the interaction profiles between enzymes (HK1 and HK2) and ligand **H2**, we observed notably stronger hydrophobic interactions of HK2 with ligand **H2** involving multiple interacting residues (Supplementary Figures S3B, D). However, we could observe significant hydrogen bonding with lesser number of interacting residues between HK1 and ligand **H2** post 100 ns of MD simulations. Notably, the reported HK2 glucosamine inhibitor exhibited a higher number of residues for interactions with HK1 active site pocket but a comparatively lesser number of interacting residues with HK2 (Supplementary Figures S3D, E).

To understand the binding affinity of the lead compound with HK1 and HK2, the Molecular mechanics/Generalized Born model and Solvent accessibility (MM-GBSA) binding energy calculations were carried out using data from MD simulations initial pose (Chen et al., 2020; Dong et al., 2021). MM-GBSA binding scores showed that the lead compound had a better affinity towards HK2 than HK1. According to the simulation results, the binding site of HK2 is notably more preferable for binding both 5HEX co-crystal ligand 604 and the proposed lead compound **H2** than the binding site of HK1. The corresponding binding free energies for the lead compound **H2** with HK1 is −23.21 kcal/mol, being 9.29 kcal/mol higher than that for the lead compound **H2** with HK2 (−32.5 kcal/mol), indicating more affinity towards HK2 (Table S3).

### 3.4 Green synthetic strategy of lead compound

Compounds **H2** and **H10** were both available from the *in-house* database. Since the initial enzyme activity assay showed marked inhibition of HK2 enzyme activity by compound **H2**, attention was given to establishing a green synthetic protocol (Zhang et al., 2021) for the prototypical lead compound **H2**. A Na^*t*^OBu-mediated approach has been made without involving any radical scavenger or transition metal for the reaction to happen through the formation of biologically important amide bond (Greenberg et al., 2000) following a direct one-pot, one-step amidation by green pathway (Sheldon, 2018; Zhang et al., 2021), avoiding column chromatography (Supplementary Figures S5–S10). A plausible mechanism is also envisaged for the same.

### 3.5 Quantitative real-time PCR

**FIGURE 2:**
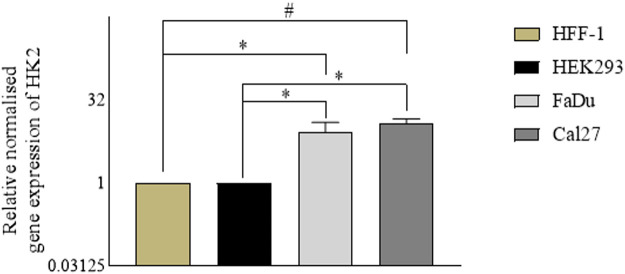
Gene expression analyses in normal cell lines (HEK293 and HFF-1) vs. cancer cell lines (FaDu and Cal27). Data are mean ± SD of three individual sets of experiments (*n* = 3). Student’s t-test *p < 0.05 and #*p* < 0.0001.

In parallel with the assessment of the anti-proliferative activity of the hit compounds, it is necessary to determine the gene expression profiles of the cancer cells (for the target genes) with respect to the normal cells. For our experiments, we used HFF-1, a normal human foreskin cell line, and HEK293, a normal human embryonic kidney cell line, with FaDu and Cal27 oral cancer cell lines. To determine the gene expression profiles of HK2, we performed quantitative real time polymerase chain reaction assays. We found HK2 was overexpressed in both FaDu and Cal27 cell lines compared to normal HFF-1 and HEK293 cells (Figure 2). The gene expression of HK2 was notably higher in Cal27 cells v/s HFF-1 and HEK293 cells, suggesting it to be a targetable protein for oral cancer therapy.

### 3.6 Glucose (2-NBDG) uptake assay as a phenotypic screen for HK2 inhibitors

**FIGURE 3:**
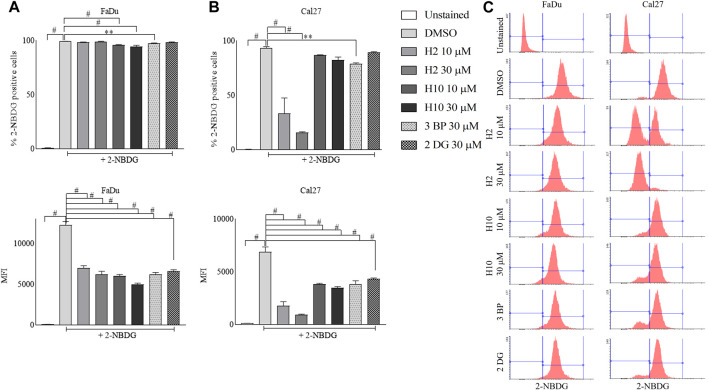
Glucose uptake assay in FaDu and Cal27 cell lines. Cells were treated **(A)** % of FaDu cell population positive for 2-NBDG and MFI of 2-NBDG in FaDu cells. **(B)** % of Cal27 cell population positive for 2-NBDG and MFI of 2-NBDG in Cal27 cells **(C)**. Histograms for 2-NBDG uptake in FaDu and Cal27 cells. Data are mean ± SD of three individual sets of experiments (*n* = 3). One-way ANOVA followed by Dunnett’s multiple comparisons test, **p < 0.01, #*p* < 0.0001.

2-[N-(7-Nitrobenz-2-oxa-1,3-diaxol-4-yl) amino]-2-deoxyglucose (2-NBDG) uptake into cells happens via glucose transporters and post uptake 2-NBDG gets phosphorylated by hexokinases. Since cancer cells represent higher glycolytic activity, the inhibition of HK2 activity in cancer cells is actively reflected in the inhibition of 2-NBDG uptake (Hung et al., 2022; Li et al., 2022). Hence, this assay was performed as a phenotypic screen to identify the inhibitors of HK2. In this assay, the MFI of intracellular 2-NBDG serves as a quantitative measure of cellular glucose uptake. The 72 h treated cultures of FaDu and Cal27 with 10 μM and 30 μM of compounds **H2**, **H10**, 3-BP and 2-DG exhibited a significant reduction of 2-NBDG MFI as represented by the left shift of histograms when compared to DMSO control. Notably, Cal27 cells demonstrated a more pronounced reduction in 2-NBDG MFI, indicating their heightened sensitivity to HK2 inhibitors. This finding is further supported by the increased % population of 2-NBDG positive cells in Cal27 cells compared to FaDu cells (Figure 3).

### 3.7 Cell cycle analyses and annexin V FITC apoptosis staining assay for HK2 inhibitors

**FIGURE 4:**
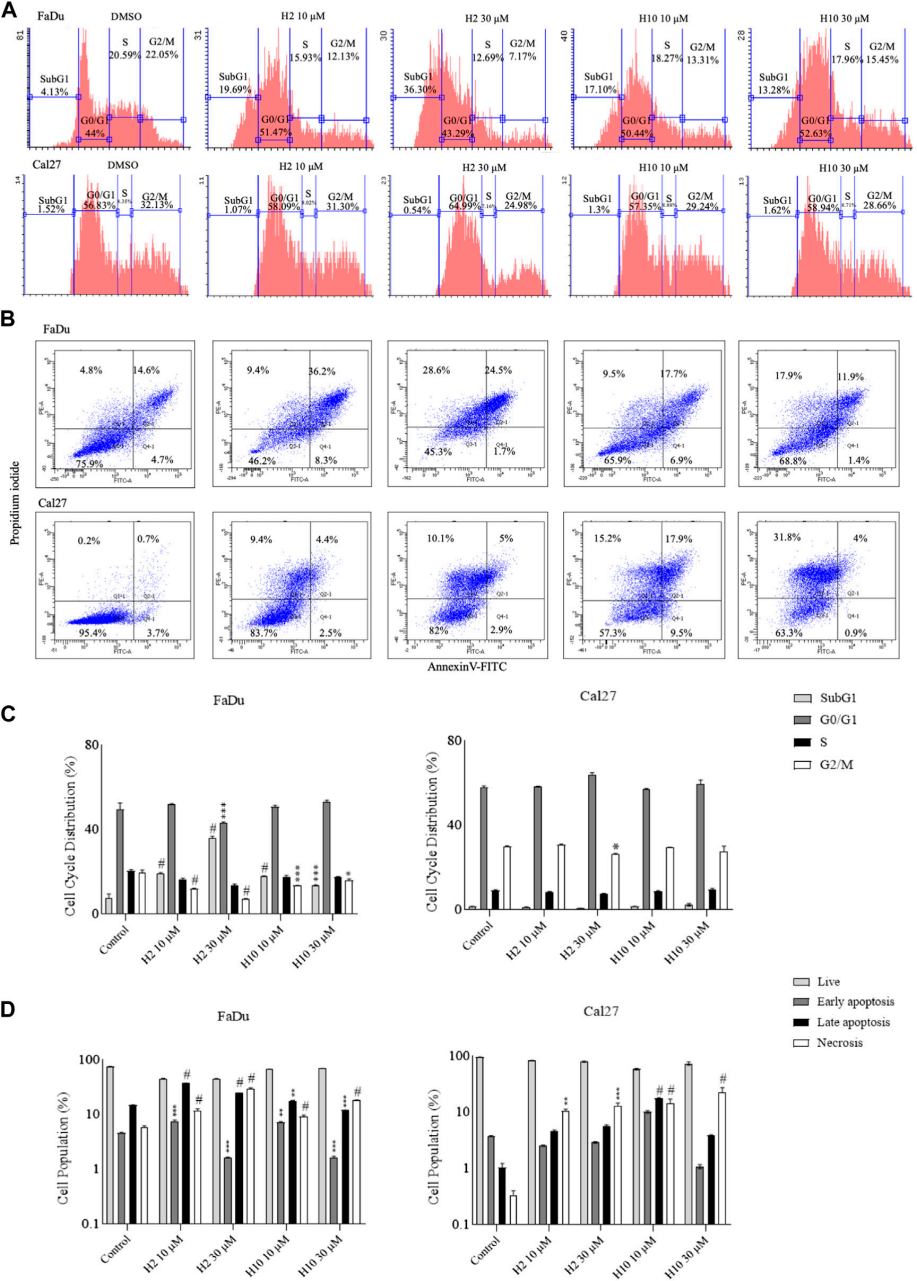
Cell cycle analyses and annexin-V FITC staining for apoptosis assay **(A)**. Area parameter histograms for cell cycle phase distribution (%) in FaDu and Cal27 cells **(B)**. % Cell population undergoing early apoptosis, late apoptosis and necrosis in FaDu and Cal27 cells **(C**, **D)**. Bar graphs represent cell cycle phase distribution and cell population (%) undergoing apoptosis. Data are mean ± SD of three individual sets of experiments (*n* = 3). One-way ANOVA followed by Dunnett’s multiple comparisons test. *p < 0.05, **p < 0.01, ***p < 0.001, #*p* < 0.0001 for treatment groups v/s control.

To determine the effect of HK2 inhibition in the progression of the cell cycle, flow cytometric analysis of compound-treated FaDu and Cal27 cells was carried out 72 h post-treatment. Cell cycle analysis is carried out to quantify the DNA content and estimate the percentages of the cell population in different cell cycle phases via propidium iodide staining. Both FaDu and Cal27 cells treated with 10 and 30 μM of compounds **H2** and **H10** showed G0/G1 phase arrest with an increase in subG1 phase population (for FaDu cells) as shown in Figure 4A. To determine the apoptosis-inducing activity of HK2 inhibitors, 10 and 30 μM of compounds **H2** and **H10** treated FaDu and Cal27 cells were stained with annexin V conjugated with FITC, followed by propidium iodide staining. The compounds exhibited late apoptotic and necrotic effects in both the cell lines after 72 h of treatment. Moreover, 10 μM of compounds **H2** and **H10** treatment was sufficient to induce apoptosis in the cell lines, as shown in Figure 4B, indicating the apoptosis-inducing activity of HK2 inhibitors.

### 3.8 Mitochondria membrane potential analysis and dissociation of MitoHK2 post HK2 inhibitor treatment

**FIGURE 5:**
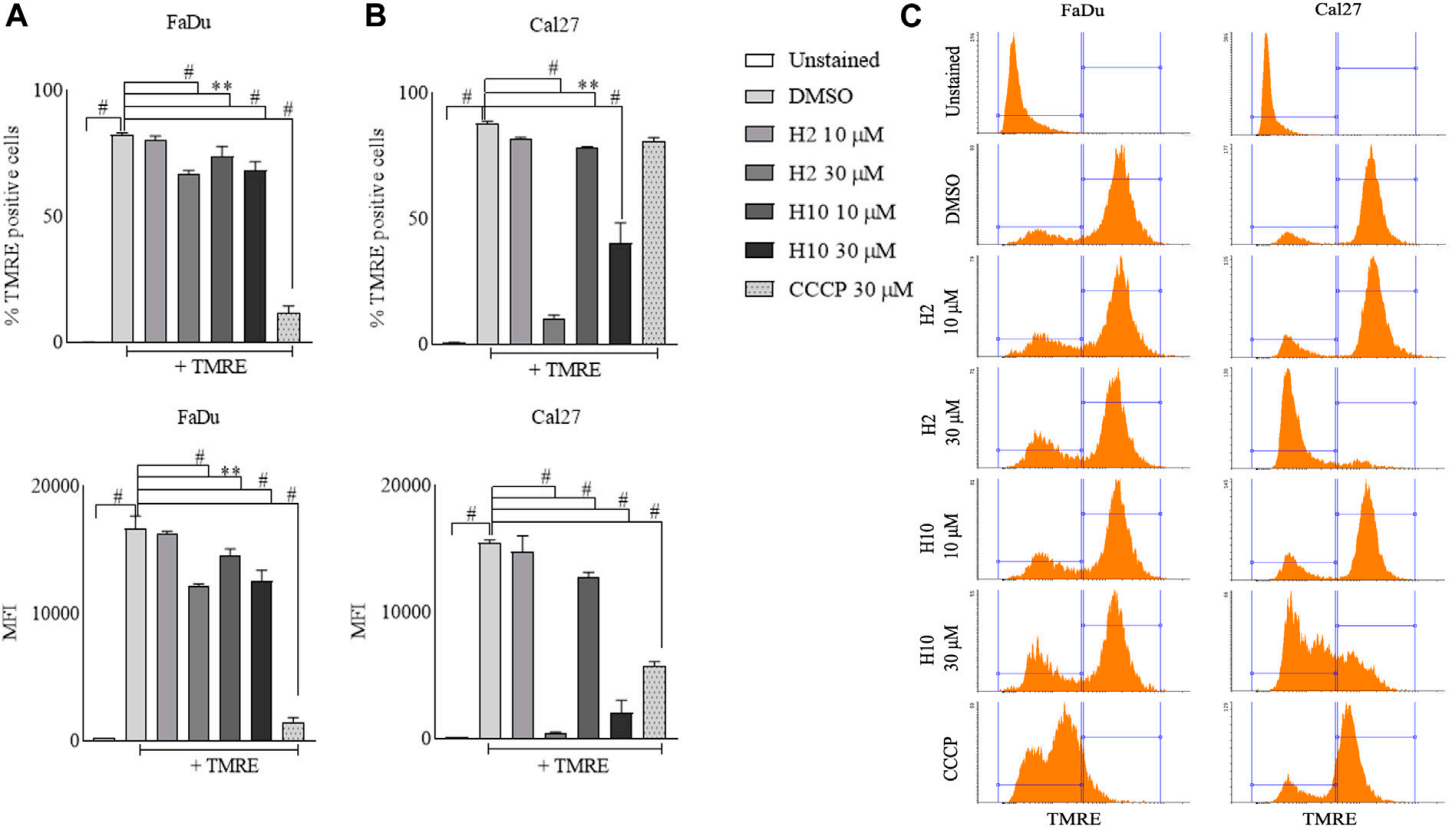
Mitochondria membrane potential analyses by TMRE uptake assay. **(A)** % TMRE positive FaDu cells and MFI of TMRE in FaDu cells post treatment. **(B)** % TMRE positive Cal27 cells and MFI of TMRE in Cal27 cells post treatment **(C)**. Histograms for TMRE uptake in FaDu and Cal27 cells. Data are mean ± SD of three individual sets of experiments (*n* = 3). One-way ANOVA followed by Dunnett’s multiple comparisons test, **p < 0.01, #*p* < 0.0001.

**FIGURE 6:**
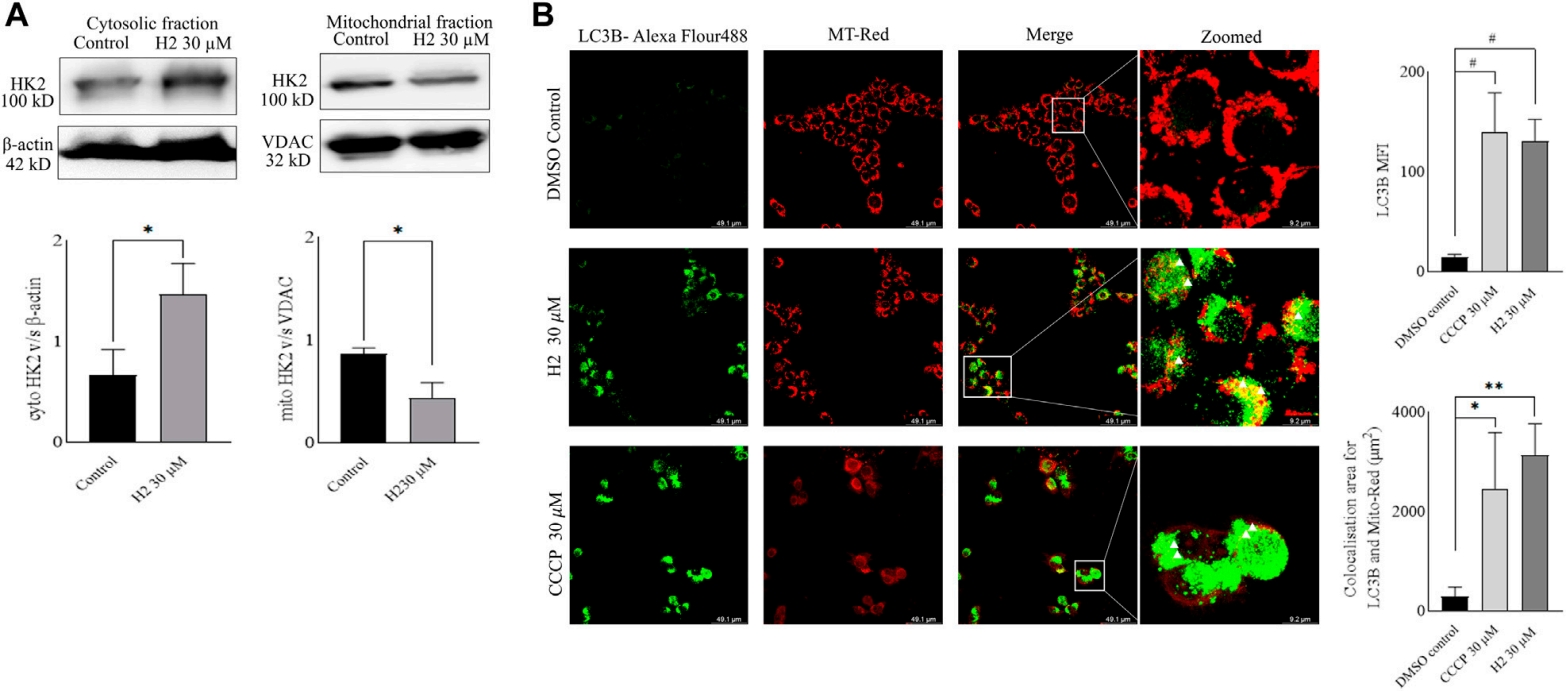
Western blot fractionation experiments and induction of mitophagy in Cal27 cells **(A)**. Expression of HK2 in the cytosolic fraction of Cal27 cells, and expression of HK2 in the mito-fractions of Cal27 cells, β-actin and VDAC are loading controls. ImageJ quantification of HK2, VDAC and β-actin bands were performed for three independent set of experiments **(B)**. LC3B and MT-Red colocalization in Cal27 cells upon treatment. DMSO control and compound treated Cal27 cells labelled with MT-Red were subjected to immunofluorescence staining using anti-LC3B antibody. The highlighted yellow dots indicate colocalised red and green colours. LAS X quantification of MFI of LC3B puncta for six independent set of experiments are represented as gray values. LAS X quantification of LC3B-Alexa flour 488 and MT-Red colocalization area from six independent sets of experiments are represented in µm^2^. Data are mean ± SD of individual sets of experiments (n = 3 for western blot fractionation experiment, *n* = 6 for immunofluorescence experiment). One-way ANOVA followed by Dunnett’s multiple comparisons test. *p < 0.05, **p < 0.01, #*p* < 0.0001.

Mitochondria utilize the oxidizable substrates to produce a membrane potential as the proton gradient (Sivandzade et al., 2019). Loss in mitochondrial membrane potential (MMP) is a direct measurement of mitochondrial stress and may cause the release of apoptotic factors leading to cell death (Zhang et al., 2017). HK2 is present in the outer mitochondrial membrane with voltage-dependent anion channel 1 (VDAC1), where it can access the newly synthesized ATP and regulate the tumor cell glucose metabolism, supporting cell proliferation, migration and resistance to apoptosis (Mazure, 2017; Chen et al., 2021). VDAC1 controls the entry and exit of ions and metabolites between cytosol and mitochondria. HK2’s association with VDAC1 maintains MMP and prevents the release of cytochrome *c.* Previous reports have mentioned that 3-BP, being an HK2 inhibitor, causes covalent modification of HK2 protein, which triggers its dissociation from mitochondria and release of apoptosis-inducing factors (AIF) from mitochondria, eventually leading to apoptosis (Rai et al., 2019). Also, HK2 malfunction has been reported to inhibit glycolysis and induce mitophagy in HCC (Li et al., 2020). To determine the change in MMP post-treatment with HK2 inhibitors, the cells were treated with 200 nM of TMRE (tetramethyl rhodamine ethyl ester, a cell-permeant cationic dye readily sequestered by active mitochondria) and flow cytometric analyses of TMRE uptake was carried out. Figure 5 shows the flow cytometric analysis of FaDu and Cal27 cells to determine the MFI of TMRE. Post 4 h treatment with HK2 inhibitors, the MFI of TMRE decreased significantly in treatment groups compared to DMSO control groups, indicating a decrease in MMP. Moreover, compound **H2** was more effective in reducing the MMP (at 30 μM concentration) compared to **H10** and CCCP. Further, the extent of MMP reduction was more prominent in Cal27 cells than in FaDu cells. Therefore, to determine the extent of mitochondrial HK2 (mitoHK2) dissociation upon inhibitor treatment, comparatively sensitive Cal27 cells were treated with 30 μM of HK2 inhibitor compound **H2**. As shown in Figure 6A, 4 h after treatment, compound **H2** induced the dissociation of mitoHK2 in Cal27 cells without altering the expression of total HK2 protein (Supplementary Figure S4A). This indicated that the dissociation of mitoHK2 was implicated in the reduction of MMP. Also, compound **H2** induced an increase in LC3B expression with the co-localization of LC3B with MitoTracker Red, suggesting an induction of LC3B mediated mitophagy (Figure 6B) and, hence, an overall efficacy of lead compound by the induction of mitochondrial toxicity in oral cancer cells was observed. Given the evidence that compound **H2** inhibits the phosphorylation of the EGFR tyrosine kinase receptor at 10 µM after 72 h of treatment, as reported earlier (Chakraborty et al., 2022), our subsequent objective was to ascertain whether the overall efficacy of **H2** resulted from the inhibition of EGFR tyrosine kinase phosphorylation or the inhibition of HK2 activity. Addressing this question, we discovered that 30 µM of **H2** does not significantly impact EGFR tyrosine kinase phosphorylation at 4 h post-treatment in Cal27 cells (Supplementary Figure S4B). This suggests that **H2** primarily functions by inhibiting HK2 enzyme activity.

### 3.9 3D spheroid culture experiments

**FIGURE 7:**
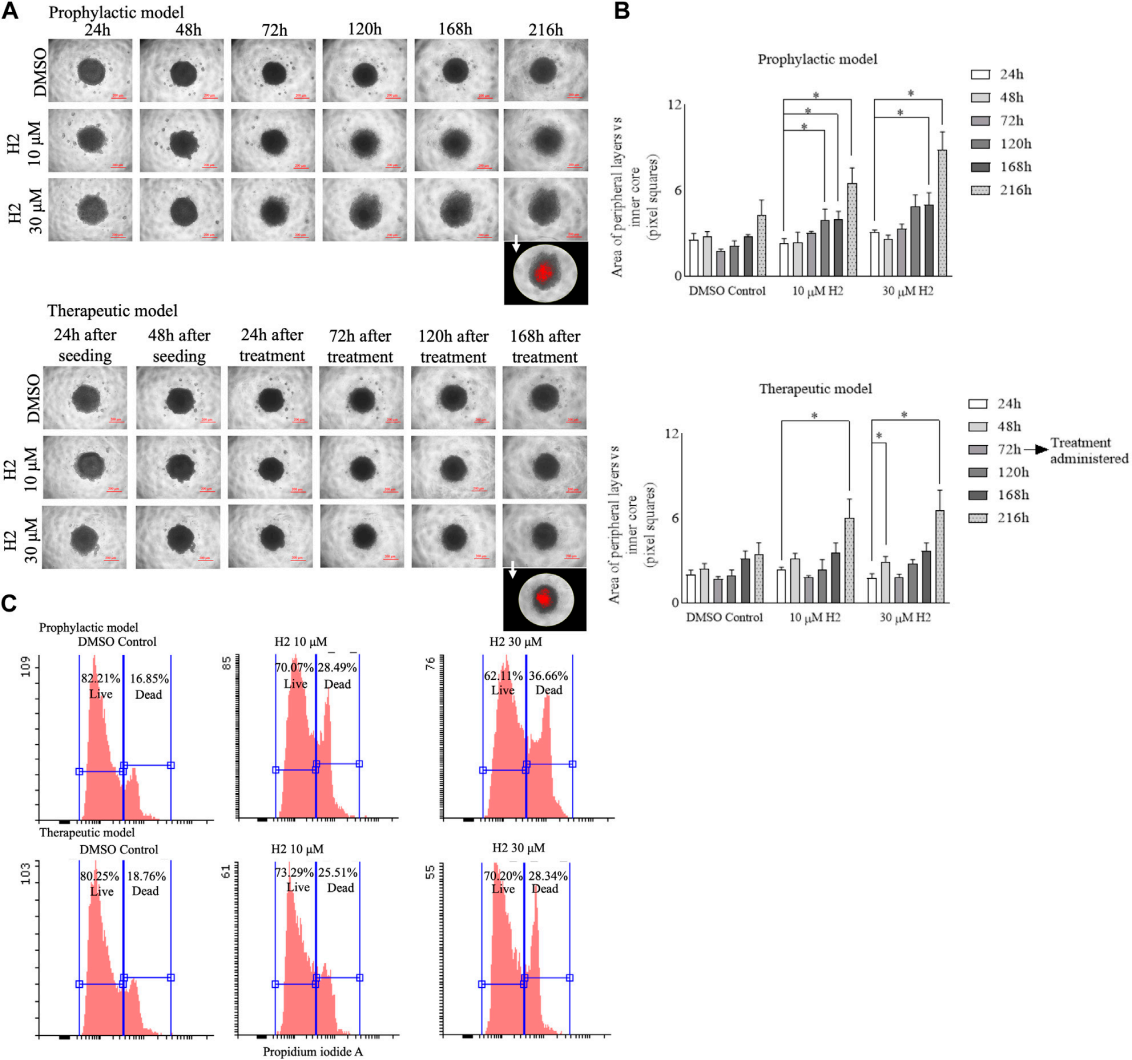
3D spheroid culture experiments of Cal27 cells **(A)**. Cal27 spheroids were grown for 9 days post seeding and images were captured. ImageJ analyses for the 8-bit images showed darker (inner core) and lighter intensity (peripheral layers) areas for each spheroid. The images in the foreground represent red highlighted inner core upon threshold adjustment **(B)**. The area of peripheral layers vs. inner core in pixel squares for the prophylactic model and for the therapeutic model **(C)**. Flow cytometric analysis for live and dead cells in spheroids after 9 days of culture for the prophylactic and therapeutic models. Data are mean ± SD of three individual sets of experiments for the area of spheroids (*n* = 3 spheroids). One-way ANOVA followed by Dunnett’s multiple comparisons test. *p < 0.05.

Cells plated in monolayer cultures might behave differently as compared to 3D cultures. The 3D cultures can better mimic the tumor development and serve as a better tool for drug screening (Zanoni et al., 2016). Therefore, spheroid cultures of Cal27 cells were prepared and treated with lead **H2**. The compound treatment (prophylactic and therapeutic) induced the reduction of spheroid growth and dissipation of cells within the 3D structure (Figure 7A). The morphology parameters were differentiated by adjusting the threshold of each spheroid image using ImageJ software. The reduction in size of the inner core compared to the peripheral layers was more in the prophylactic model than in the therapeutic model (Figure 7B). Flow cytometric analysis of Cal27 spheroids for live/dead cells using propidium iodide staining was also carried out at the end of the study, showing an increase in the population of dead cells upon **H2** treatment (Figure 7C).

## 4 Discussion

Oral squamous cell carcinoma (OSCC) is a common type of oral malignancy resulting in disfiguration and functional impairments, with a substantial impact on quality of life of patients (Feller et al., 2021; Tan et al., 2023). The enhanced glycolysis in cancer cells even in the presence of oxygen has emerged as a hallmark of cancer progression (Liberti and Locasale, 2016). Hexokinase 2 (HK2), a key enzyme involved in the first step of glycolysis, plays a pivotal role in driving this metabolic shift by facilitating the phosphorylation of glucose to glucose-6-phosphate (Patra et al., 2013). Notably, HK2 is overexpressed in various cancers, including oral cancer, where it contributes to tumor growth, metastasis and therapeutic resistance as evidenced by Kaplan Meier survival analysis from TCGA (Figure S1). Previous reports have demonstrated the efficacy of glucosamine derivatives in inhibiting the HK2 activity, highlighting HK2 as an attractive therapeutic target (Lin et al., 2016). By understanding the intricate interplay between dysregulated metabolism and oncogenic signaling pathways, researchers aim to devise innovative strategies for disrupting tumor metabolism and sensitizing cancer cells to conventional therapies (Tan et al., 2023). Thus, elucidating the molecular mechanisms underlying HK2 dysregulation holds profound implications for the development of targeted therapies tailored for oral cancer.

The utilization of structure-based and ligand-based drug design strategies has revolutionized the field of drug discovery, offering innovative avenues for repurposing existing drugs and designing novel therapies (Castleman et al., 2022; Chakraborty et al., 2022). In our study, we capitalized on these strategies to target HK2. By leveraging the available crystal structures of HK2 protein complexed with co-crystal ligands, we gained crucial insights into the substrate (glucose) binding site, facilitating rational drug design efforts (Figure 1A). In light of the structural insights from the HK2 crystal structure, we focused on a hypothesis driven approach, leveraging energy-based pharmacophores (EPs) generated from co-crystal ligands, to guide the rational design of HK2 inhibitors. By generating a diverse array of hypothesis derived from the 4 available ligands, we culminated in the validation of 27 hypothesis, which showed the robustness of our predictive models in filtering out active molecules from a pool of decoy ligands (Figure 1B). Subsequently, the validated EPs served as valuable tools for screening a comprehensive ligand library comprising US-FDA approved drugs and compounds synthesized in house, thereby enriching our pool of potential HK2 inhibitors.

Furthermore, we employed high-throughput virtual screening (HTVS), standard precision (SP) and extra-precision (XP) docking to prioritize candidate compounds with favorable binding interactions at the active site of HK2. Our virtual screening campaign yielded a promising set of hits, representing potential HK2 inhibitors worthy of further evaluation (Table S2).

The evaluation of the anti-proliferative activity of the inhibitors and their HK2 enzyme inhibitory activity provides crucial insights into their therapeutic potential against oral cancer. Hits derived from the structure-based drug design strategy were subjected to a comprehensive assessment using FaDu and Cal27 oral cancer cells. Treatment with the compounds for 72 h at varying concentrations revealed their dose-dependent effects on cell proliferation. Notably, the standard HK2 inhibitor 2-deoxy glucose (2-DG) exhibited a GI50 of 77.56 µM in Cal27 cells and demonstrated limited efficacy against FaDu cells. In contrast, 3-bromo pyruvate (3-BP) displayed its potency in inhibiting cell proliferation of FaDu and Cal27 cells. The differential sensitivity of FaDu and Cal27 cells to the different compounds, as indicated by their distinct GI50 values, underscores the importance of considering cell line-specific responses in therapeutic development (Table S2). Importantly, compounds **H2** and **H10**, previously identified for their EGFR phosphorylation inhibitory potential, exhibited promising IC50 values against HK2, further highlighting their therapeutic relevance.

In parallel, we conducted molecular dynamics (MD) simulations to elucidate the dynamic behavior of lead compounds in complex with HK2 protein over extended time scales. MD simulations provided invaluable insights into the stability of lead compound-HK2 complexes, shedding light on their binding modes and conformational dynamics. Specifically, the crystal ligand RMSD exhibited slightly higher variation in the HK2 simulations than lead compound suggesting stable and optimal fitting of lead compound in the binding pocket (Figure S2 B and D). Conversely, HK1 simulations displayed higher RMSD fluctuations indicating a less favorable binding mode and reduced stability of the lead compound at the active site, highlighting a potential in achieving selective inhibition (Figure S2 A and C). Furthermore, analysis of interaction profiles revealed stronger and frequent hydrophobic interactions between HK2 and lead compound, underscoring its preferential binding affinity for HK2 over HK1 (Figure S3). To quantitatively assess the binding affinity of the lead compound for HK1 and HK2, MM-GBSA binding energy calculations were performed using data from the initial pose of MD simulations. The MM-GBSA binding scores corroborated the superior binding affinity of the lead compound for HK2 compared to HK1, with more favorable binding free energies observed for HK2, highlighting its potential as a selective HK2 inhibitor (Table S3).

The assessment of gene expression profiles is imperative for understanding the molecular mechanisms underlying the observed antiproliferative effects of the hit compounds in oral cancer cells. To elucidate the role of HK2 in cancer progression, we conducted qRT-PCR assays to compare its expression levels in oral cancer cell lines (FaDu and Cal27) with those in normal human foreskin (HFF-1) and embryonic kidney (HEK293) cells lines. Our findings revealed a significant upregulation of HK2 expression in both FaDu and Cal27 cell lines compared to HFF-1 and HEK293 cells, suggesting its potential as a targetable protein (Figure 2). Interestingly, the HK2 expression levels between FaDu and Cal27 cells were found to be comparable, indicating a similar degree of HK2 overexpression in these 2 cell lines. In Figure 3, we present evidence illustrating the distinct response of FaDu and Cal27 cells to treatment with HK2 inhibitor, revealing their differential sensitivity to HK2 inhibitor treatment based on their glucose uptake efficiency. Despite the comparable HK2 expression between FaDu and Cal27 cells, Cal27 cells exhibited a significantly greater reduction in glucose uptake following treatment with **H2** compared to FaDu cells. This observation suggests that while both cell lines exhibit similar levels of HK2 expression, Cal27 cells may possess distinct molecular characteristics that render them more susceptible to HK2 inhibition by **H2**. Although in FaDu cell line, **H10** treatment marked significant changes in glucose uptake than **H2** treatment, it is essential to delve deeper into the differences between these cell lines. The underlying mechanisms driving this differential response warrant further investigation to elucidate the specific factors contributing to the sensitivity to HK2 inhibitors. In summary, while both FaDu and Cal27 cells exhibit elevated HK2 expression levels characteristic of cancer cells, their response to HK2 inhibitors differ significantly. The observed disparities in glucose uptake sensitivity underscore the importance of considering cell line-specific responses when evaluating the efficacy of HK2-targeted therapies. If Cal27 cells indeed exhibit a higher dependence on HK2-mediated glycolysis for their survival, targeting HK2 may represent a more effective therapeutic strategy for Cal27-derived oral cancers. Future research aimed at elucidating the molecular mechanisms governing these differences may facilitate the development of personalized treatment strategies tailored to individual patient profiles and tumor characteristics (Zhang et al., 2021). It is important to mention here that in our previous study (Chakraborty et al., 2022), we investigated the effects of HK2 inhibitor treatment on glucose transporter (GLUT) expression levels in FaDu and Cal27 cell lines. Our results indicated that the treatment with the HK2 inhibitor at the concentration of 10 µM did not result in significant changes in GLUT expression levels, which indicate that the observed changes in glucose uptake following treatment with lower concentration of HK2 inhibitor is likely attributable to the direct inhibition of HK2 enzyme activity rather than alterations in GLUT expression.

The impact of HK2 inhibition on cell cycle progression and apoptosis induction was also investigated in FaDu and Cal27 cell lines treated with compounds **H2** and **H10**. Flow cytometric analysis was employed to evaluate the effects of these compounds on distribution of cells across different phases of the cell cycle. Treatment with 10 and 30 µM concentrations of HK2 inhibitors resulted in a notable accumulation of cells in the G0/G1 phase, indicative of cell cycle arrest, accompanied by an increase in the subG1 phase population (Figure 4A). This observed G0/G1 phase arrest suggests a potential disruption of cell proliferation dynamics following HK2 inhibition (Figure 4C), highlighting the anti-proliferative effects of these compounds. Furthermore, to assess the apoptotic activity of HK2 inhibitors, FaDu and Cal27 cells treated with 10 and 30 µM concentrations of HK2 inhibitors were subjected to annexin V-FITC and propidium iodide staining followed by flow cytometric analysis. The results revealed a significant induction of late apoptosis and necrosis in both cell lines following 72 h of treatment. Notably, treatment with 10 µM of these compounds was sufficient to trigger apoptosis in both cell lines (Figure 4B). Interestingly, a comparative analysis of apoptosis and necrosis levels between FaDu and Cal27 revealed that FaDu cells exhibited higher levels of apoptosis and necrosis compared to Cal27 cells following treatment with **H2** and **H10**. This observation suggests that FaDu cells may be more sensitive to the apoptotic effects of HK2 inhibitors than Cal27 cells (Figure 4D).

The mitochondria play a pivotal role in cellular energy metabolism, generating ATP through oxidative phosphorylation (OXPHOS) while maintaining mitochondrial membrane potential (MMP). HK2 localized on the outer mitochondrial membrane (OMM) in association with VDAC regulates glucose metabolism and supports various cellular processes including proliferation, migration, and apoptosis resistance (Mazure, 2017; Chen et al., 2021). Notably, HK2’s interaction with VDAC helps maintain MMP and inhibits the release of apoptotic factors such as cytochrome *c*. Additionally, dysregulation of HK2 function has been linked to glycolysis inhibition and induction of mitophagy, further emphasizing its role in cancer cell survival and metabolism. In this study, we investigated the effect of HK2 inhibition on MMP in FaDu and Cal27 oral cancer cells. Treatment with HK2 inhibitors resulted in a significant reduction in MMP, indicative of mitochondrial stress and potential induction of apoptosis. Particularly, compound **H2** exhibited superior efficacy in reducing MMP compared to **H10** and CCCP, with a more pronounced effect observed in Cal27 cells (Figure 5). Further, analysis revealed that compound **H2** induced the dissociation of mitoHK2 in Cal27 cells without altering total HK2 protein expression (Figure S4A), suggesting a direct link between mitoHK2 dissociation and MMP reduction (Figure 6A). The decision to utilize a concentration of 30 µM for mitoHK2 dissociation was based primarily on a dose-response study for the analysis of MMP, which indicated that 10 µM of compound did not induce a significant reduction in MMP. As such, a 30 µM concentration was selected to ensure a robust and measurable effect on mitoHK2 at the 4 h time point, which is distinct from the 72 h time point utilized for other assays. While the previous experiments have demonstrated significant effects of 10 µM of HK2 inhibitor, we opted for a higher concentration in the context of mitoHK2 dissociation to ensure a more pronounced and consistent response in this specific assay. Moreover, compound **H2** treatment led to an increase in expression of LC3B puncta and co-localization with MitoTracker Red, indicating the induction of LC3B-mediated mitophagy and overall mitochondrial toxicity in oral cancer cells (Figure 6B). Importantly, despite its known inhibition of EGFR tyrosine kinase phosphorylation, compound **H2** did not significantly impact EGFR phosphorylation at 4 h post-treatment, highlighting its primary mode of action through HK2 enzyme inhibition rather than EGFR signaling modulation (Figure S4B).

The use of 3D cultures, such as spheroids, offers several advantages over traditional monolayer cultures. Firstly, the 3D cultures better mimic the *in vivo* tumor microenvironment, including cell-cell interactions, nutrient gradients and extracellular matrix components, thereby providing a more physiologically relevant model for studying drug response (Vinci et al., 2012). Additionally, 3D cultures allow for the assessment of drug penetration and distribution within the tumor mass, which is critical for evaluating the efficacy of anti-cancer agents in heterogenous cell populations. Moreover, the ability to monitor long-term drug effects and evaluate complex phenotypic changes, such as morphological alterations and cell-death dynamics, makes 3D cultures a valuable tool for preclinical drug development (Vinci et al., 2012). Therefore, in this study, we employed spheroid cultures of Cal27 cells to assess the efficacy of lead compound **H2**. The treatment with **H2**, both prophylactically and therapeutically, induced a noticeable reduction in spheroid growth and led to the dispersion of cells within the 3D structure (Figure 7A). To quantify these morphological changes, we adjusted the threshold of each spheroid using ImageJ software and observed a more significant reduction in the size of the inner core compared to the peripheral layers, particularly in the prophylactic model (Figure 7B). Flow cytometric analysis of Cal27 spheroids for live/dead cells using propidium iodide staining further supported the efficacy of **H2** treatment, revealing an increase in the population of dead cells at the end of the study (Figure 7C). Overall, our findings demonstrate the effectiveness of compound **H2** in inhibiting spheroid growth and inducing cell death in Cal27 3D cultures, underscoring its potential as a promising therapeutic candidate for oral cancer. The use of 3D culture models enhances our understanding of drug response mechanisms and facilitates the identification of novel anti-cancer agents with improved efficacy and selectivity.

## 5 Conclusion

The present paper discusses a multi-pharmacophore-based high-throughput virtual screening campaign conducted using Schrodinger software modules for drug discovery. Various HK2 proteins were employed in this screening approach, which yielded promising hit compounds with diverse scaffolds (pharmacophores). Subsequent assessments of the efficacy of these compounds against HK2 were conducted using different cell-based assays. Among the tested compounds, a benzothiazole-carboxamide compound (referred to as **H2**) and a benzothiazole-methoxyphenyl urea compound (referred to as **H10**) demonstrated inhibition of HK2 enzyme activity. Intriguingly, these two compounds had been previously reported as EGFR-TK inhibitors for oral cancer therapy by our same research group. The convergence of hit compounds from the previous and current studies suggests an expanded scope for identifying prototypical lead compounds against cellular kinases using a multiple pharmacophore-based screening approach. Additional observations include HK2 inhibitors induce mitoHK2 dissociation and LC3B-mediated mitophagy. Western blot fractionation experiments and confocal co-localisation experiments were conducted to elucidate the mechanism of action of lead compound induced cell death. 3D spheroid culture experiments showed the effectiveness of HK2 inhibitor in reducing Cal27 spheroid growth. In conclusion, this work provides a foundation for tool compounds to study HK2 mediated effects in oral cancer. Further studies are required to determine the *in vivo* effect of the lead compound.

## Data Availability

The original contributions presented in the study are included in the article/Supplementary Materials, further inquiries can be directed to the corresponding authors.
