# Supplementary material for: Targeting hexokinase 2 for oral cancer therapy: structure-based design and validation of lead compounds
Source: Front Pharmacol. 2024 Mar 11;15:1346270. doi: 10.3389/fphar.2024.1346270 (PMC10961359; doi:10.3389/fphar.2024.1346270)
Supplement: Supplementary file 1 [file DataSheet1.docx]

**Supporting Information**

**Targeting Hexokinase 2 for Oral Cancer Therapy: Structure-Based Design and Validation of Lead Compounds**

Purbali Chakraborty^1,4^, Syeda Lubna^2^, Shouvik Bhuin^3^, Deepika K^1^, Manab Chakravarty^3^, Trinath Jamma^2,4^*, Perumal Yogeeswari^1,4^*

^1^Department of Pharmacy, Birla Institute of Technology and Science, Pilani Hyderabad Campus, Hyderabad, 500078, Telangana, India

^2^ Department of Biological Sciences, Birla Institute of Technology and Science, Pilani Hyderabad Campus, Hyderabad, 500078, Telangana, India

^3^Department of Chemistry, Birla Institute of Technology and Science, Pilani Hyderabad Campus, Hyderabad, 500078, Telangana, India

^4^Cancer Research Group, Centre for Human Diseases Research, Birla Institute of Technology and Science, Pilani, Hyderabad Campus, Hyderabad, 500078, Telangana, India

*Corresponding Authors: Perumal Yogeeswari, pyogee@hyderabad.bits-pilani.ac.in

Trinath Jamma, trinath@hyderabad.bits-pilani.ac.in

**Table of Contents**

| **Topics** | **Page/s** |
| --- | --- |
| **Table S1**. Enrichment scores of e-pharmacophore subset features. | 3 |
| **Table S2**. The GI50 of hits derived from virtual screening. | 4 |
| **Table S3**. Calculated MM-GBSA binding energy. | 7 |
| **Table S4**. Selectivity index of leads. | 8 |
| **Figure S1**. Kaplan Meier Overall Survival Analyses in Head & Neck Cancer for HK2. | 9 |
| **Figure S2**. Protein-Ligand RMSD for 100 ns molecular dynamic simulations (HK1 and HK2 proteins) with lead **H2** and co-crystal ligands. | 10 |
| **Figure S3**. Interacting residues of HK1 and HK2 proteins with the lead compound **H2** and co-crystal ligands. | 11 |
| **Figure S4**. Western blot analyses for HK2 and pEGFR expression in Cal27 lysates 4h post treatment with **H2**. | 12 |
| **Figure S5**. Scheme of green synthesis of the lead molecule **H2** (N-(3-hydroxy-4methoxybenzyl)benzo[d]thiazole-2-carboxamide). | 13 |
| Synthetic procedure and characterizations | 13 |
| **Figure S6**. ^1^H NMR of compound **H2** | 14 |
| **Figure S7**. ^13^C NMR of compound **H2** | 14 |
| **Figure S8.** IR of compound **H2** | 15 |
| **Figure S9.** HRMS spectra of compound **H2** | 15 |
| **Figure S10**. Plausible mechanism | 16 |

**Table S1**. Enrichment scores of e-pharmacophore subset features.

| **Hypothesis ID^a^** | **Subset Pharmacophore^b^** | **EF1%** | **BEDROC α=20.0** | **BEDROC α= 160.9** |
| --- | --- | --- | --- | --- |
| 1 | A3A4D9D10 | 100 | 0.932 | 0.874 |
| 2 | A3A4D11D7 | 100 | 0.874 | 0.981 |
| 3 | A3A4D7D10 | 100 | 0.607 | 0.895 |
| 4 | A3A4D11 D10 | 50 | 0.782 | 0.962 |
| 5 | A3A4D9D7 | 100 | 0.716 | 0.904 |
| 6 | A3A4D9D11 | 100 | 0.544 | 0.782 |
| 7 | A3A2D9D10 | 100 | 0.540 | 0.611 |
| 8 | A3A2D11D7 | 50 | 0.334 | 0.566 |
| 9 | A3A2D7D10 | 50 | 1 | 1 |
| 10 | A3A2D11D10 | 50 | 0.874 | 0.981 |
| 11 | A3A2D9D7 | 100 | 0.597 | 0.887 |
| 12 | A3A2D9D11 | 100 | 0.782 | 0.962 |
| 13 | A3A2D11D8 | 50 | 1 | 1 |
| 14 | A3A2D9D8 | 100 | 1 | 1 |
| 15 | A3A2D7D8 | 100 | 0.782 | 0.962 |
| 16 | A5A6D9D10R15 | 100 | 1 | 1 |
| 17 | A5D9R15 | 100 | 0.381 | 0.887 |
| 18 | A5A6D9D11R15 | 100 | 0.852 | 0.980 |
| 19 | A6D11R15 | 100 | 0.448 | 0.905 |
| 20 | A5A6D9D10 | 100 | 0.874 | 0.981 |
| 21 | A5A6D10R15 | 100 | 1 | 1 |
| 22 | A2A3D7D8 | 100 | 1 | 1 |
| 23 | A2A3D7D9 | 100 | 0.932 | 0.990 |
| 24 | A2A3D7D10 | 50 | 0.632 | 0.910 |
| 25 | A2A3D7 | 100 | 0.932 | 0.99 |
| 26 | A2A3D9 | 50 | 0.333 | 0.841 |
| 27 | A2A3D10 | 50 | 0.539 | 0.892 |

a The hypotheses 1 to 15 represents 2NZT, hypotheses 16 to 21 represents 5HEX, hypotheses 22 to 27 represents 5HG1; b The subsets were derived by combining pharmacophores of original ligand. The above table represents only the subset pharmacophores derived from the main pharmacophore.

**Table S2**. The GI50 of hits derived from virtual screening.

| **Compound** | **GI50 for FaDu (µM)**^a^ | **GI50 for Cal27 (µM)**^a^ | **% inhibition of HK2 enzyme activity at 50 µM** |
| --- | --- | --- | --- |
| 1 (3-Bromo pyruvic acid)   | 23.85 ± 1.01 | 36.36 ± 4.02 | 88.2 % |
| 2   | 66.20 ± 4.55 | >100 | No inhibition |
| 3   | 5.28 ± 1.07 | 1.6 ± 4.9 | No inhibition |
| 4   | 36.87 ± 2.86 | 12.85 ± 4.1 | No inhibition |
| 5   | 73.15 ± 2.74 | >100 | No inhibition |
| 6   | 37.33 ± 1.82 | 12.49 ± 7.2 | No inhibition |
| 7   | 60.03 ± 1.11 | >100 | No inhibition |
| 8   | 30.80 ± 1.98 | 0.97 ± 3.2 | No inhibition |
| 9   | >100 | 4.8 ± 2.9 | 2.4% |
| 10   | 50.96 ± 0.45 | 25 ± 2.41 | No inhibition |
| 11 (**H10**)   | 65.92 ± 0.79 | 28.29 ± 2.25 | 82.8% (IC50 = 13.89 ± 0.5 µM) |
| 12   | 82.67 ± 0.89 | 88.25 ± 2.36 | No inhibition |
| 13   | 39.35 ± 0.40 | 25.60 ± 3.15 | 47.3% |
| 14 (**H2**)   | 63.06 ± 1.08 | 21.70 ± 1.5 | 87% (IC50 = 2.94 ± 0.2 µM) |
| 15 (2-Deoxy Glucose)   | >100 | 77.56 ± 4.3 | Not tested |

^a^ All the data are represented in mean *±* SEM

**Table S3**. Calculated MM-GBSA binding energy.

| **Protein-PDB ID** | **Ligand** | **ΔG^MM-GBSA^ (kcal/mol)** |
| --- | --- | --- |
| HK1 - 1CZA | Co-crystal (glucose) | -40.18 |
| HK1 - 1CZA | **H2** | -23.21 |
| HK1 – 1CZA | Glucosamine derv. (Ligand 604) | -34.69 |
| HK2 - 5HEX | Co-crystal (Ligand 604) | -111.54 |
| HK2 - 5HEX | **H2** | -32.5 |

**Table S4**. Selectivity index of leads.

| **Leads** | **CC50 in HFF1 (µM)** | **Selectivity index (FaDu)^a^** | **Selectivity index (Cal27)^a^** |
| --- | --- | --- | --- |
| **H10** | 42.66±2.36 | 0.65 | 1.51 |
| **H2** | >100 | >1.6 | >4.6 |

^a^ Selectivity index is calculated as CC_50_/GI_50_


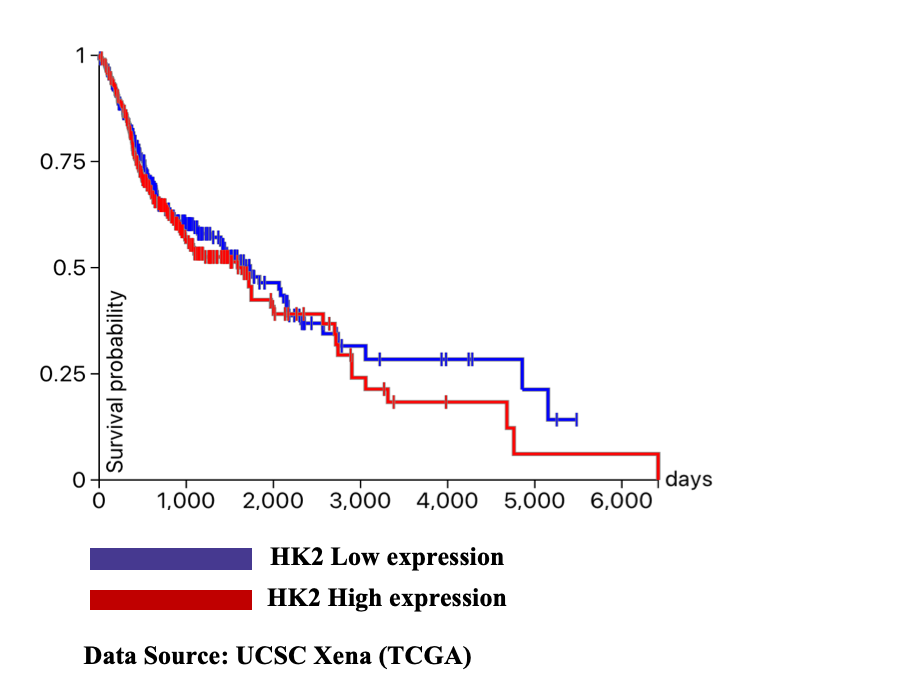


**Figure S1**. Kaplan Meier Overall Survival Analyses in Head & Neck Cancer for HK2.


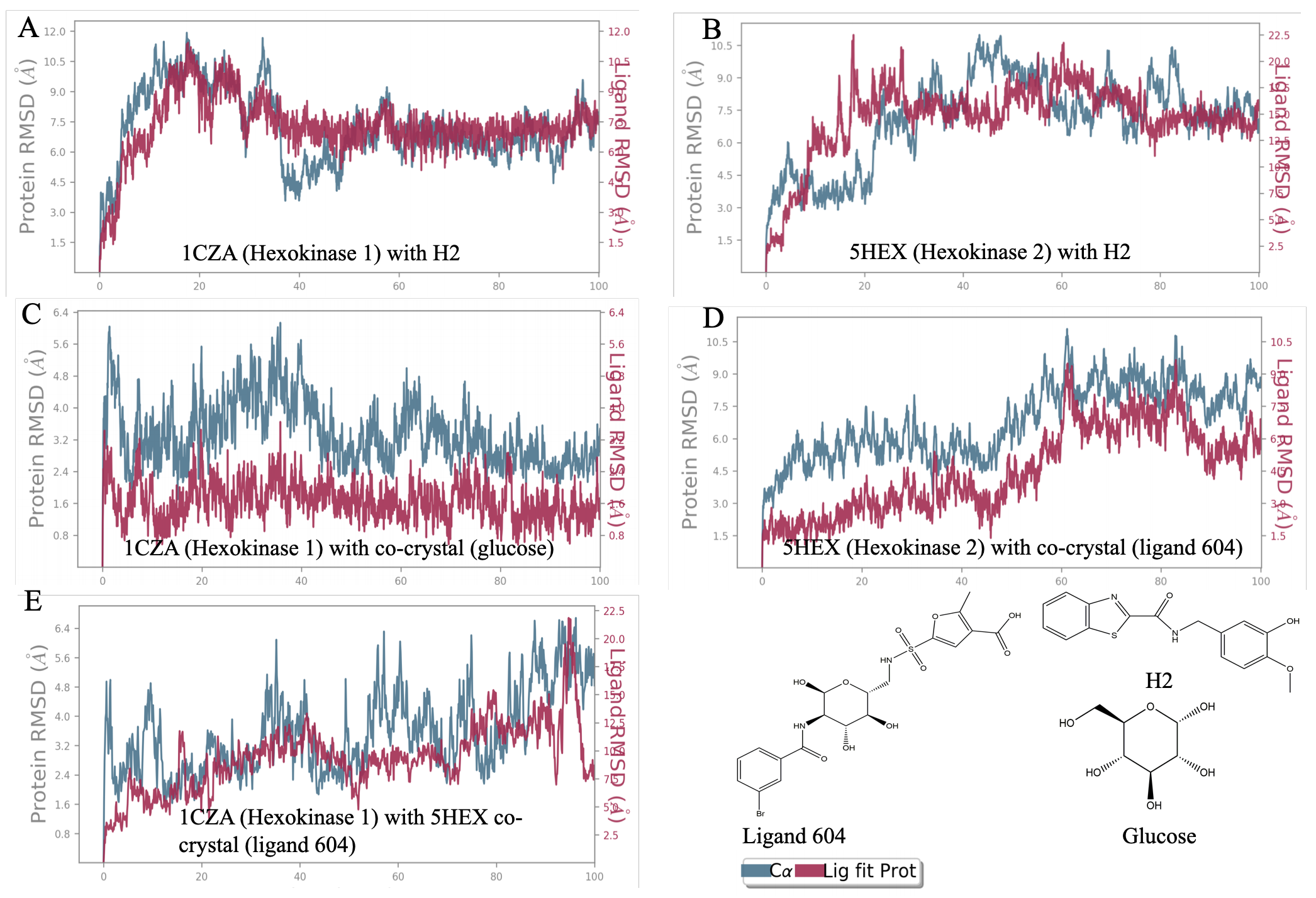


**Figure S2**. Protein-Ligand RMSD for 100 ns molecular dynamic simulations (HK1 and HK2 proteins) with lead **H2** and co-crystal ligands.


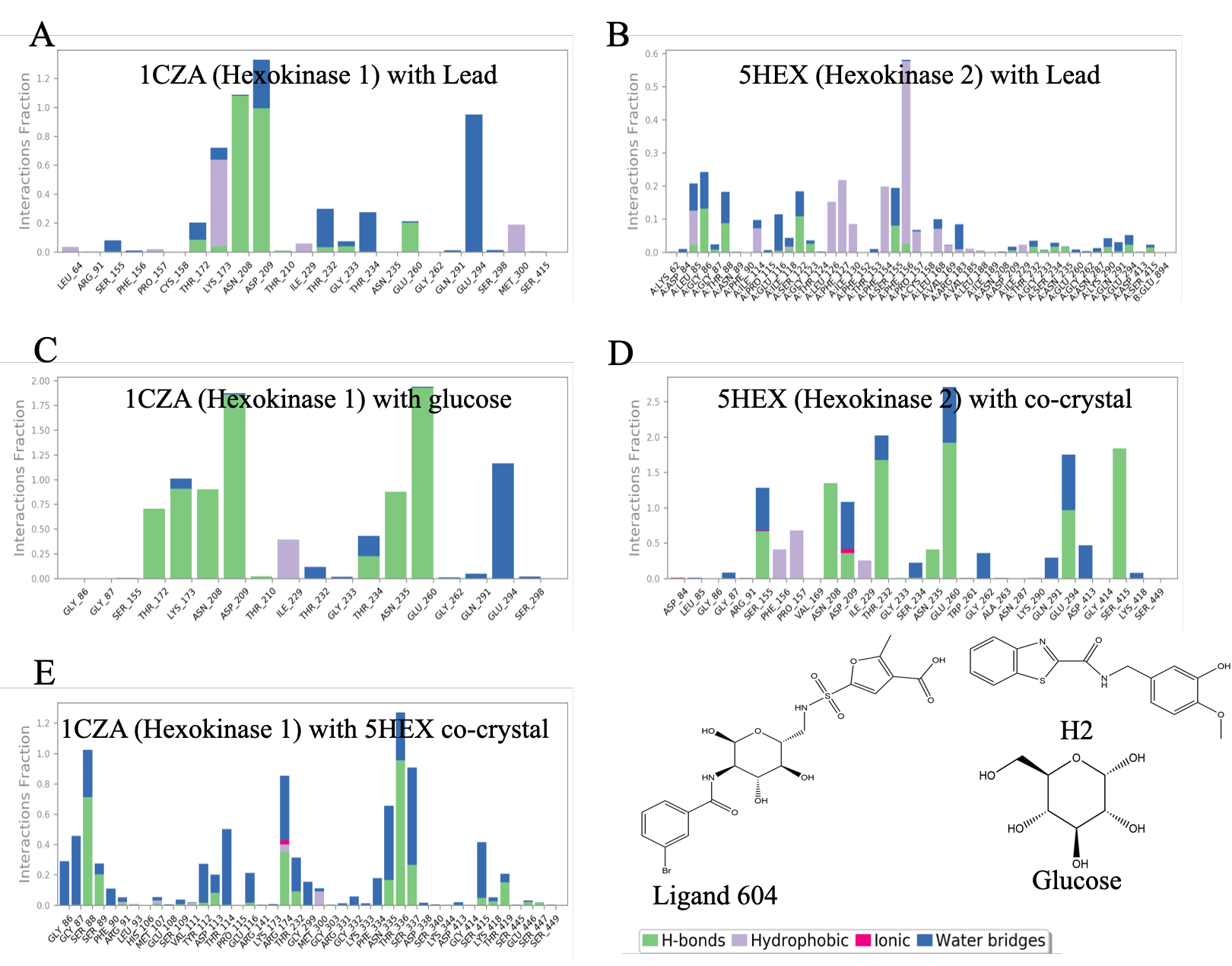


**Figure S3**. Interacting residues of HK1 and HK2 proteins with the lead compound **H2** and co-crystal ligands.


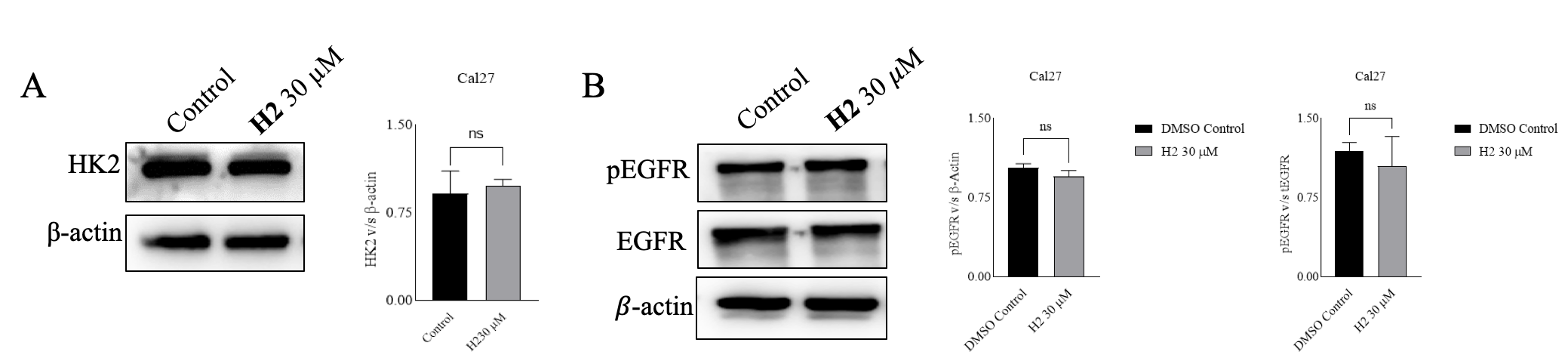


**Figure S4**. Western blot analyses for HK2 and pEGFR expression in Cal27 lysates 4h post treatment with **H2**.


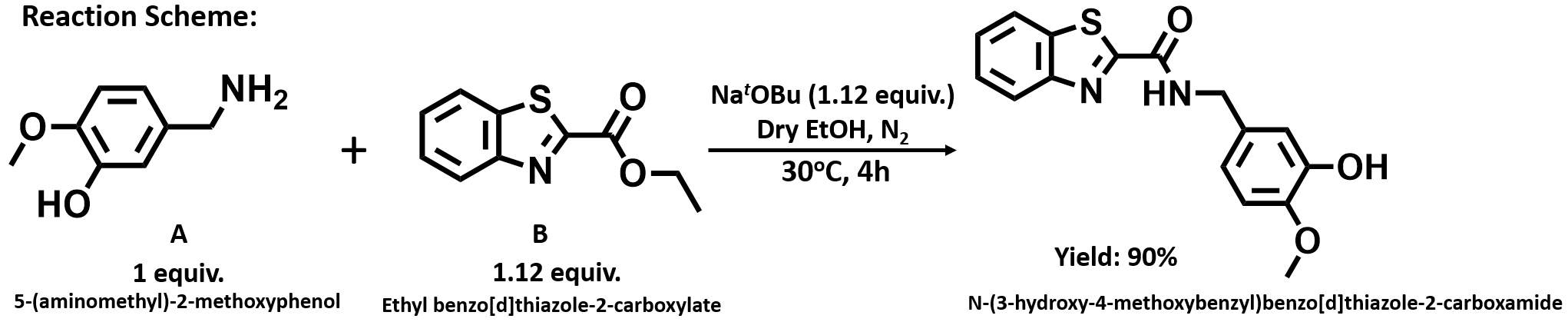


**Figure S5**. Scheme of green synthesis of the lead molecule **H2** (N-(3-hydroxy-4methoxybenzyl)benzo[d]thiazole-2-carboxamide).

**Synthetic procedure and characterizations:**

In a two-neck round bottom flask 5-(aminomethyl)-2-methoxyphenol (5.353 mmol, 0.82 gm, 1 equiv) was taken, and ethyl benzo[d]thiazole-2-carboxylate (5.995 mmol, 1.25 gm, 1.12 equiv) was taken to be vacuum dried for 5-10 mins. Later, dry EtOH (30 ml) was added and stirred for 5 mins under an N_2_ environment. Next, under this N_2_ environment, Na*^t^*OBu (5.995 mmol, 0.58 gm, 1.12 equiv) was added to the reaction mixture and stirred for 4h at 30^o^C. Over completion of the reaction, the product started to precipitate. The reaction mixture was then allowed to remain undisturbed and the product precipitated out. The upper layer was decanted, and the product was further washed with fresh ethanol and dried subsequently to receive a white solid product (*R_f_* = 0.3, in 30% EtOAc into hexane (v/v), yielding 90% (1.51 gm). IR (KBr, cm^-1^): 3466, 3335, 1676, 1546, 1515, 1440, 1326, 1273, 1130, 1024. M.p.: 108-110^o^C; NMR spectroscopy: ^1^H NMR (400 MHz, CDCl_3_, 25^o^C, ⸹): 7.98-7.95 (m, 1H), 7.92-7.89 (m, 1H), 7.61 (*br.*, s, 1H), 7.49-7.40 (m, 2H), 6.90-6.75 (m, 3H), 5.59 (*br.*, s, 1H), 4.53 (d, *J*= 4Hz, 2H), 3.82 (s, 3H). ^13^C NMR (100 MHz, CDCl_3_, 25^o^C, ⸹): 163.84, 159.7, 152.9, 146.3, 145.9, 137.1, 130.6, 126.8, 126.7, 124.3, 122.4, 119.8, 114.3, 110.8, 56.1, 43.6. Mass spectrometry: HRMS (ESI) m/z: calcd. for C_16_H_14_N_2_O_3_S 314.0725, found: 315.0802 [M+H]^+^.


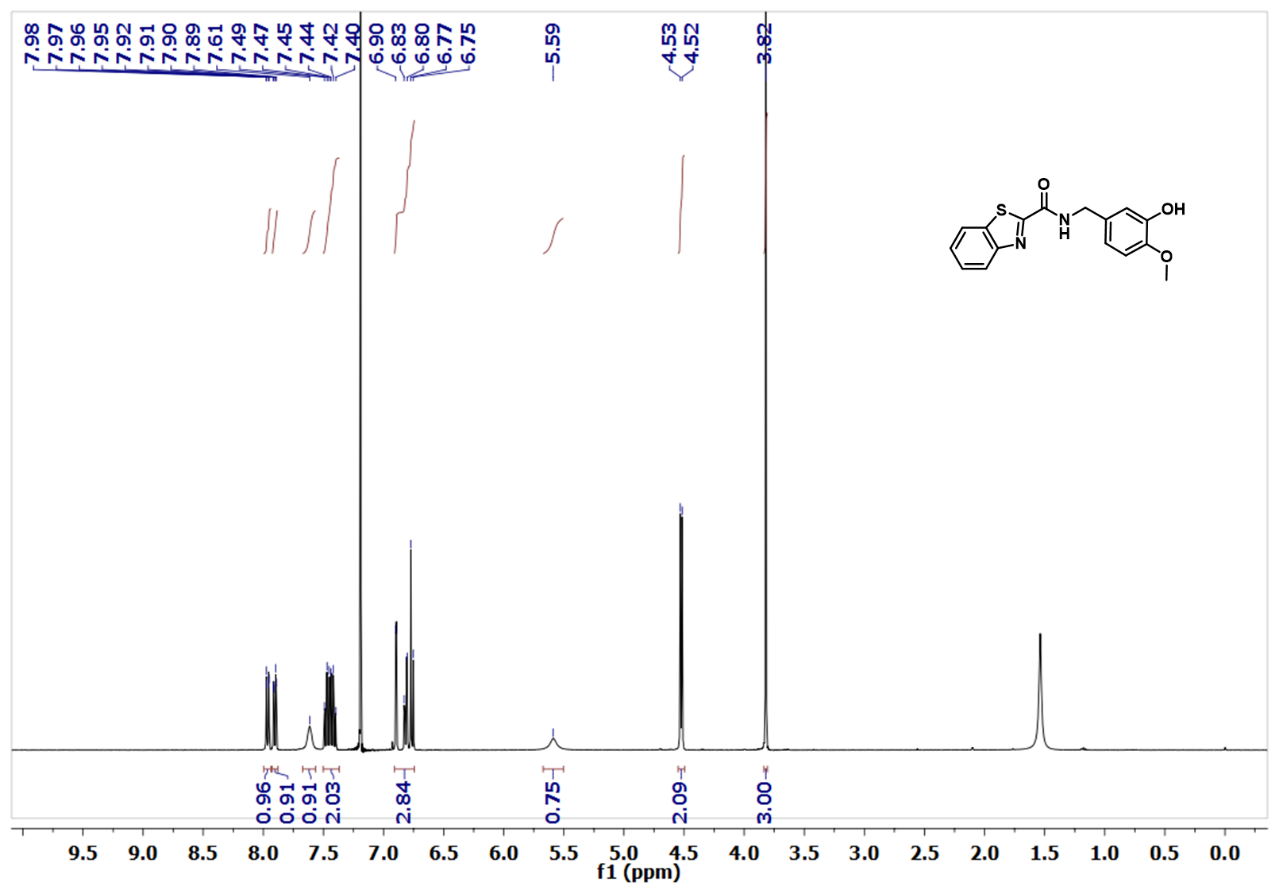


**Figure S6**. ^1^H NMR of compound **H2** (water peak is there at ~ 1.5 ppm).

**
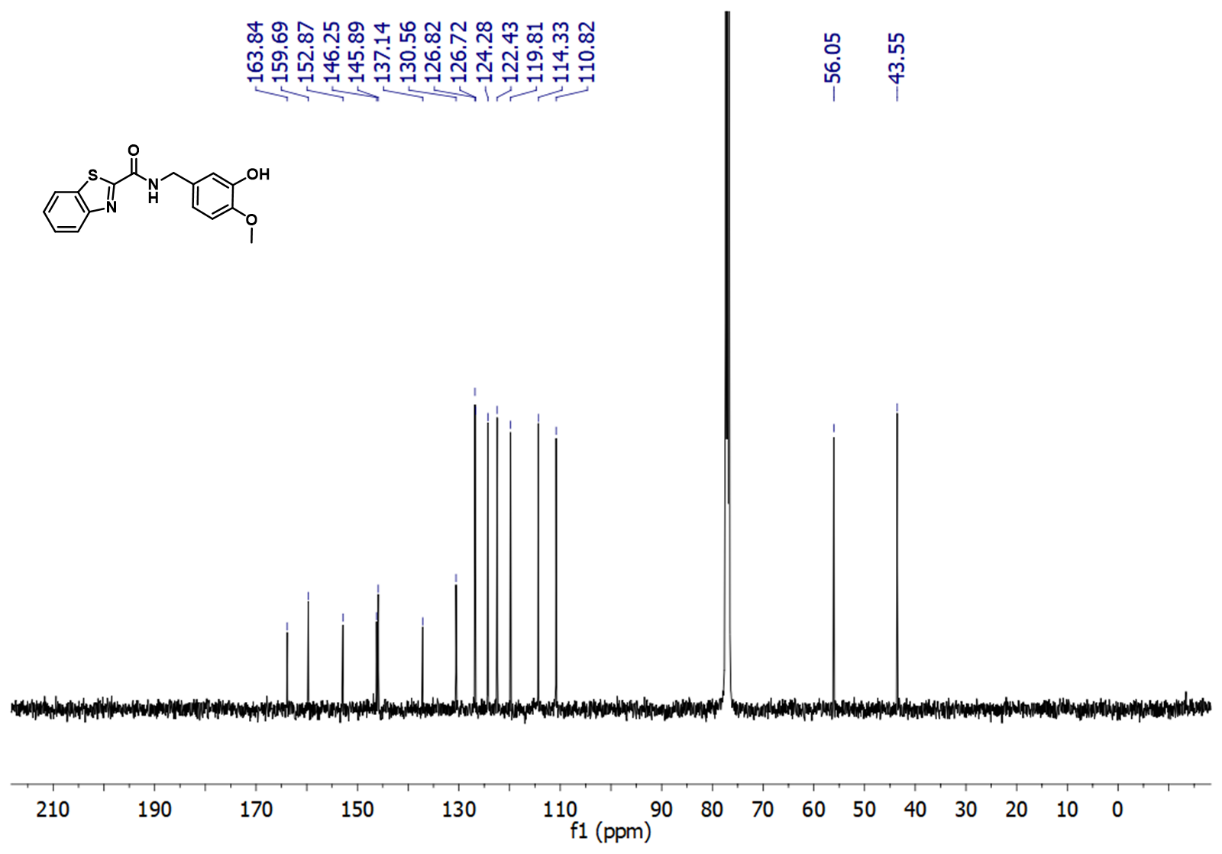
**

**Figure S7**. ^13^C NMR of compound **H2**.


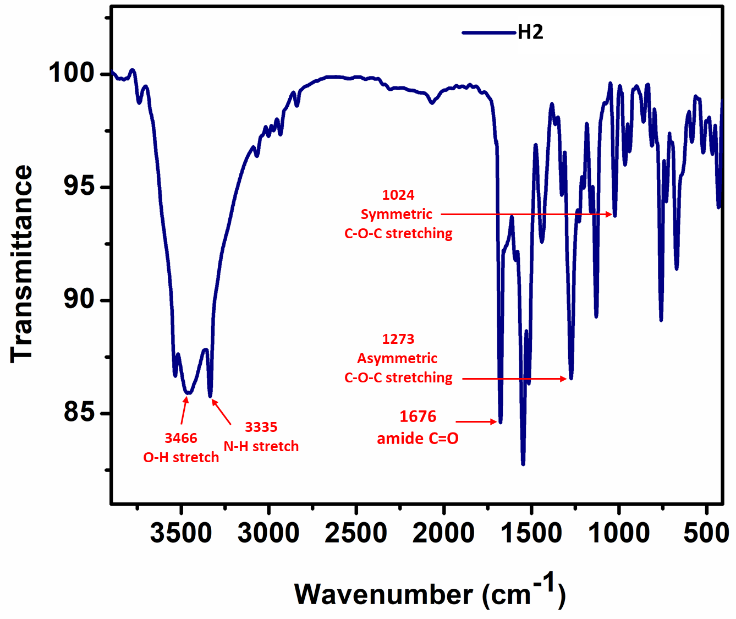


**Figure S8**. IR of compound **H2**.

**
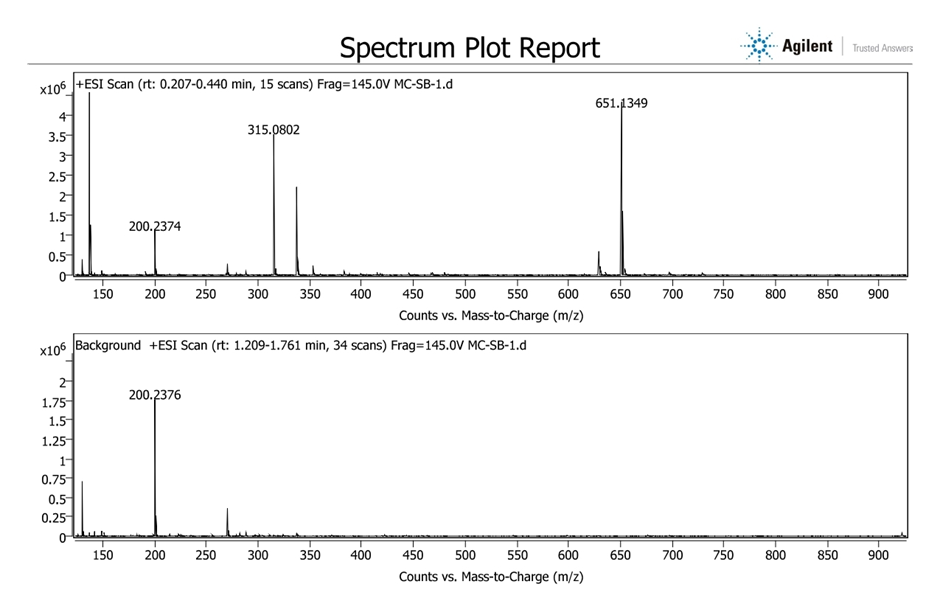
**

**Figure S9**. HRMS spectra of compound **H2**.

**Plausible mechanism:**

A Na*^t^*OBu-mediated green synthetic approach has been envisaged to produce the bioactive lead molecule **H2**, avoiding the involvement of any radical scavenger or transition metal. It must be noted that sustainable protocol on direct amidation of unactivated methyl esters has been reported earlier (1). Herein, we started with ethyl benzo[d]thiazole-2-carboxylate as an ethyl ester and 5-(aminomethyl)-2-methoxyphenol as our amine. We utilized a strong non-nucleophilic base Na*^t^*OBu, and the amine acts as the nucleophile in the presence of the non-nucleophilic base. Another possible role of the base would be to activate the carbonyl carbon and make it more prone to undergo a nucleophilic attack by the amine through the formation of a chelate intermediate (Intermediate II) (Figure S10).


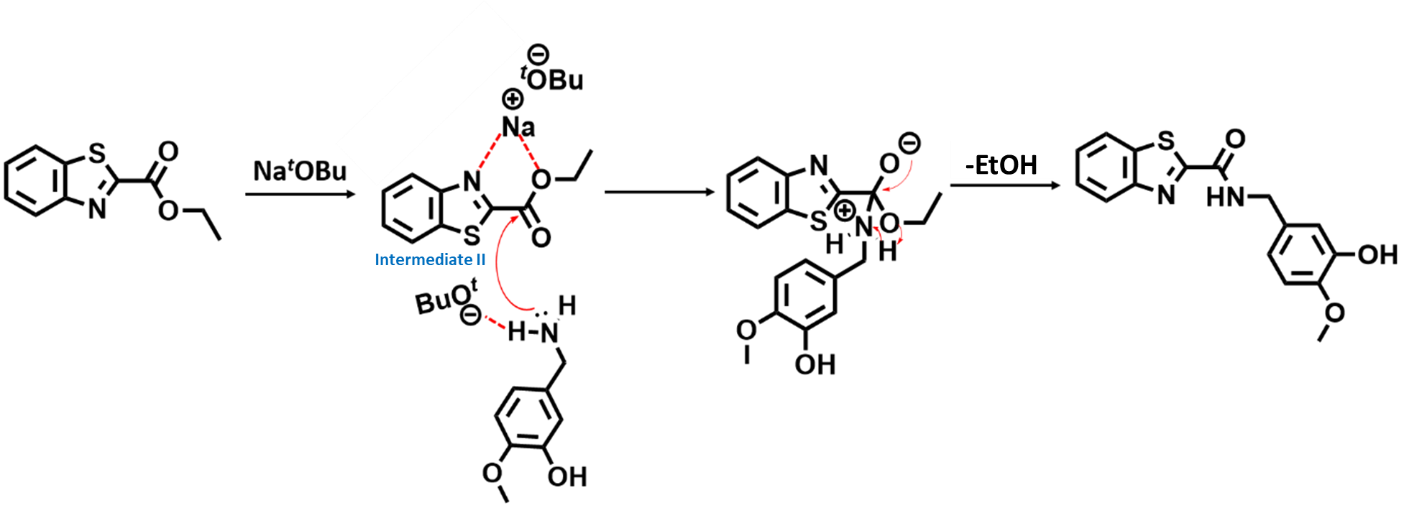


**Figure S10**. A plausible mechanism for the Na*^t^*OBu-mediated green synthetic approach.

1. Zhang, R., Yao, W.Z., Qian, L., Sang, W., Yuan, Y., Du, M.C., Cheng, H., Chen, C. and Qin, X., 2021. A practical and sustainable protocol for direct amidation of unactivated esters under transition-metal-free and solvent-free conditions. Green Chemistry, 23(11), pp. 3972-3982.

**Note**: The NMR spectra were recorded at ambient temperature (ca. 20◦C) in CDCl_3_ solution and calibrated against the characteristic solvent peaks (7.26 ppm in 1H, 77.0 ppm in 13C NMR). The chemical shifts were reported in ppm by denoting the multiplicities as s (singlet), d (doublet), and m (multiplet). An FT-IR spectrometer (FT/IR-4200, Jasco) was used to record the IR spectra of the samples. Solid samples were mixed with KBr to form pellets to record the spectra.
